# Arthroscopic debridement followed by intra‐articular injection of micro‐fragmented adipose tissue in patients affected by knee osteoarthritis: Clinical results up to 48 months from a prospective clinical study

**DOI:** 10.1002/jeo2.70144

**Published:** 2025-01-17

**Authors:** Andrea Antonio Maria Bruno, Giuseppe Anzillotti, Massimo De Donato, Marco Basso, Jacopo Tamini, Paolo Dupplicato, Elizaveta Kon, Berardo Di Matteo, Enrico Arnaldi

**Affiliations:** ^1^ IRCCS Humanitas Research Hospital Rozzano Milan Italy; ^2^ Department of Biomedical Sciences Humanitas University Pieve Emanuele Milan Italy; ^3^ Department of Traumatology Orthopaedics and Disaster Surgery, Sechenov University Moscow Russia

**Keywords:** adipose‐derived stem cells, arthroscopy, knee, mesenchymal stem cells, osteoarthritis

## Abstract

**Purpose:**

Micro‐fragmented adipose tissue is emerging as a promising option for the treatment of various diseases including knee osteoarthritis (OA), though clinical trials are often limited by short follow‐up periods. Our aim was to evaluate the safety and clinical outcomes of an arthroscopic debridement followed by a single injection of micro‐fragmented adipose tissue in patients affected by knee OA.

**Methods:**

From 2016 to 2020, patients affected by knee OA were enroled. Micro‐fragmented adipose tissue was obtained through the Lipogems® kit and intraarticularly injected after an arthroscopic debridement. Visual analogue scale for pain, Tegner score, Knee Injury and Osteoarthritis Outcome score subscales variations were assessed from baseline to 3, 6, 12, 24 and 48 months of follow‐up.

**Results:**

Forty‐one patients were evaluated up to 6 months of follow‐up, 39 patients up to 24 months of follow‐up and 38 patients up to 48 months of follow‐up. Three underwent knee replacement surgery during the time of the study. All the clinical scores analyzed achieved statistically significant changes up to the last follow‐up.

**Conclusions:**

A single intra‐articular knee injection of micro‐fragmented adipose tissue following arthroscopic debridement is able to provide significant clinical benefits in patients affected by knee OA up to 4 years of follow‐up.

The present clinical study was registered on clinicaltrials.gov (no. NCT06545266).

**Level of Evidence:**

Level IV case series.

**Abbreviations**
FDA: Food and Drug Administration
KL: Kellgren–Lawrence
KOOS: Knee Injury and Osteoarthritis Outcome
MSC: mesenchymal stem cell
OA: osteoarthritis
QoL: quality of life
VAS: visual analogue scale

## INTRODUCTION

Osteoarthritis (OA) is a whole joint disease which very commonly affects the knee joint [3]. After low back pain, knee OA is considered the second musculoskeletal disorder in disability‐adjusted life years for the adult population [36, 37]. Its burden has a dramatic impact on the healthcare system, with esteemed direct and indirect costs approximately up to 2.5% of one country's domestic product [16, 23, 24].

Conservative treatments, such as oral anti‐inflammatories or intra‐articular viscosupplementation, may be useful as symptomatic relief in earlier stages [18, 21], but there is no effect on disease progression. The ultimate treatment for knee OA still consists of total joint arthroplasty, though it still suffers from biological complications (i.e., infections) and mechanical alterations (i.e., material wearing, loosening or mobilization) [5, 20]. Given these limitations, new conservative approaches are emerging to postpone as much as possible the prosthetic implantation. Cartilage repair procedures are able to provide clinical benefits in patients suffering from well‐delimited cartilage lesions; however, they are not indicated for patients suffering from diffuse knee OA [2, 14, 17]. Corticosteroid injections may be indicated in cases of diffuse diseases, even though their effects could range from detrimental to disease‐modifying [7]. Orthobiologics have seen their fortune in the last 20 years as the most innovative therapies available on the market. In particular, mesenchymal stem cells (MSCs) are being investigated as the holder for the anti‐inflammatory effect inside the joint, able to attenuate the OA symptoms, hence recently deserving the title of ‘medicinal signalling cells’ [11, 12, 27]. Furthermore, the ‘secretome effect’ also includes the intraarticular release of numerous growth factors (i.e., HIF—hypoxia‐inducible factor, bFGF—basic fibroblast growth factor, IGFs—insulin‐like growth factors and VEGF—vascular endothelial growth factor), stimulating chondrogenesis and MSCs proliferation itself [26, 32]. Recent evidences on animals suggest that their role may not only be limited to a symptomatic or functional impact but they could also serve as disease‐modified drugs [9, 34]. Recent clinical trials have confirmed these results in humans, demonstrating reduction of pain and increased functional improvement [33, 42]. Interestingly, a recent trial by Cattaneo et al. proved a significant improvement in KOOS score in patients affected by knee OA, undergone chondral shaving associated with micro‐fragmented adipose tissue knee injection [13]. Nonetheless, this evidence is limited by short follow‐up, which limits the impact of such findings. Accordingly, the aim of the present study was to evaluate the reduction in pain, effects on functional scores and safety of an arthroscopic debridement followed by a single injection of micro‐fragmented adipose tissue in patients affected by knee OA at longer follow‐ups.

## MATERIALS AND METHODS

### Patients' selection

The present clinical study was registered on clinicaltrials.gov (no. NCT06545266). From 2016 to 2020, a total of 41 patients with different degrees of knee OA were prospectively enroled for this study. The internal ethical committee of the Humanitas Research Hospital, Milan, Italy approved the present study (study no. 2219). A written informed consent was obtained from each patient prior to enrolment.

Inclusion criteria were: age >18 years, patients affected by radiologically confirmed knee OA according to Kellgren–Lawrence (KL) classification (Grades I–IV), not responsive to previous standard conservative treatments (i.e., physical therapy, exercise, oral pharmacological treatment and intra‐articular injections of corticosteroids/hyaluronic acid), with symptoms in the index knee persisting for at least three consecutive months at the moment of study enrolment.

Exclusion criteria were as follows: recent traumatic events involving the index joint, chondromatosis, intra‐articular injections of any type in the previous 6 months, malignancy, pregnancy, patients on anticoagulant therapy or suffering from coagulopathies and related disorders, systematic disorders affecting the joints (i.e., rheumatoid arthritis). Enroled patients underwent a knee radiograph (antero‐posterior, Rosenberg and lateral projections) as well as magnetic resonance imaging before the procedure to stage knee OA and evaluate potential intra‐articular loose bodies or diseases different from knee OA (e.g., tumours or focal cartilage lesions).

### Surgical procedure

Patients were placed in a supine position and Betadine was used to treat the abdomen. Anaesthesia was performed using disposable blunted cannulas with a modified Klein solution (a mixture of 250 mL of saline solution [4°C], 50 mL of lidocaine [2%] and 1 mL of epinephrine, 1:1000). Two skin incisions of approximately 0.5 cm were performed on both sides of the abdomen and the solution was injected in the subcutaneous fat tissue. Tissue harvesting was performed, connecting the VacLock® vacuum syringe to an aspiration blunt cannula. Approximately the same quantity of tissue was harvested from each side of the patient's abdomen, with a minimum of 100 ml. All procedures were performed by at least two physicians the first physician prepared and used the Lipogems® ortho kit, and the other performed a debridement arthroscopic procedure on the selected knee in order to exclude the presence of other causes of pain (e.g., loose bodies and meniscal flaps). The debridement procedure consisted of the removal of eventual chondral flaps and the regularization of eventual degenerative meniscal lesions.

Once the aspiration process was completed, approximately 100 mL of lipoaspirate was processed each time in the standard 225 mL device. The Lipogems® ortho kit is made by a core, a simple disposable device that progressively reduces in size the clusters of adipose tissue (from 1 to 3.5 mm to clusters of approximately 0.2–0.8 mm) while at the same time eliminating oil and blood residues. After the appropriate tissue preparation, the final Lipogems® product was ready to be collected in 10 mL syringes from the upper opening and ready for its knee intra‐articular injection at the level of the superolateral edge of the patella. This process required approximately 20 min. and the final product volume obtained was roughly 25%–30% of the initial tissue that was harvested. Skin portals were sutured and elastic body belt was applied and maintained for 10 days. Patients were usually discharged the same day of the procedure with a post‐operative pain management protocol (Paracetamol 1000 mg 1 tablet three times a day for 5 days and Ibuprofen 600 mg 1 tablet twice a day for 2 days) and a thromboembolic prophylaxis protocol (low molecular weight heparin for 1 week). Crutches were suggested for the first two days, and full weight bearing was allowed from the first post‐operative day.

### Clinical assessments

#### Primary outcome

Visual analogue scale (VAS) for pain variation from baseline to 3, 6, 12, 24 and 48 months of follow‐up.

#### Secondary outcomes

The Tegner score and Knee Injury and Osteoarthritis Outcome (KOOS) score subscales assess variations from baseline to 3, 6, 12, 24 and 48 months of follow‐up, the safety of the procedure and the failure rate.

Moreover, results from clinical scores were also stratified according to three age groups (<50, between 50 and 60 and >60 years).

Data were also stratified and analyzed with respect to the OA grading classification KL scale, dividing patients into mild OA (K‐L1).

### Failures

Treatment failure was defined as any surgical treatment performed on the index knee during the study period.

### Statistical analysis

Data were described as number and percentage, if categorical, or mean and standard deviation if continuous. Change of SCALE within time was explored with multilevel mixed‐effects regression. Estimates were also adjusted by age and KL scale, and stratified by age divided in decades, and KL, dichotomized as Grades I and II versus Grades III and IV.

All analyses were made with Stata17 programme. A *p < *0.05 was considered statistically significant.

## RESULTS

A total of 41 patients were enroled in the present study. 41 patients were evaluated up to 6 months of follow‐up, 39 patients up to 24 months of follow‐up and 38 patients up to the 48 months (final) of follow‐up. Twenty‐six patients were males and 15 were females. Five patients were KL Grade I, 16 patients were KL Grade II, 18 patients were KL Grade III and 2 patients were KL Grade IV. Characteristics of the included patients are listed in Table 1.

**Table 1:** Demographic characteristics of the included patients.

	Value (*N* = 41)
Age	61 ± 9
Gender (female)	15/41
BMI	28 ± 4.8
Index knee (right)	25/41
Kellgren–Lawrence grade	
I II III IV	5/41 16/41 18/41 2/41
Index knee (right)	25/41
Arthroscopic procedures performed	
Debridement alone Debridement + degenerative meniscal flaps removal Debridement + loose body removal	36/41 3/41 2/41

Abbreviation: BMI, body mass index.

**Figure 1:**
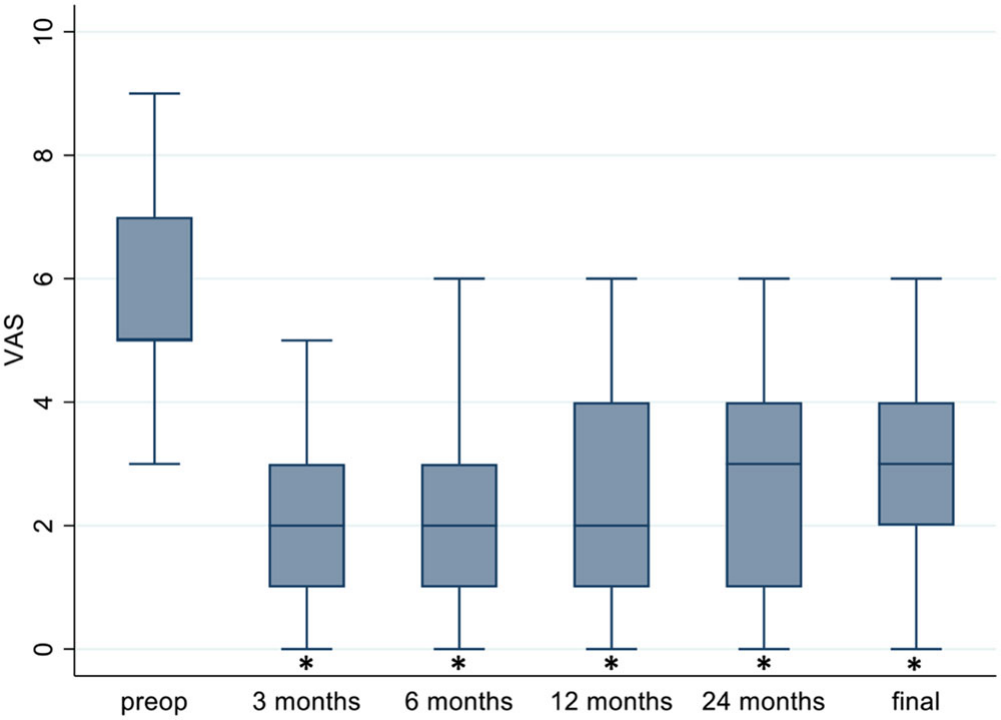
Evaluation of visual analogue scale (VAS) for pain at 3, 6, 12 and 48 months of follow‐up. On the plot, values from top to bottom are maximum, 75th percentile, median, 25th percentile and minimum.*Statistically significant (*p* < 0.05) compared to baseline.

The average VAS pre‐operatively was 5.8 ± 1.63. At 3 months, there was a statistically significant decrease to 2.22 ± 1.4 (*p* < 0.05) and remained stable at 6 and 12 months to then slightly increase to an average of 2.89 ± 1.64 at the end of the study (Figure 1).

**Figure 2:**
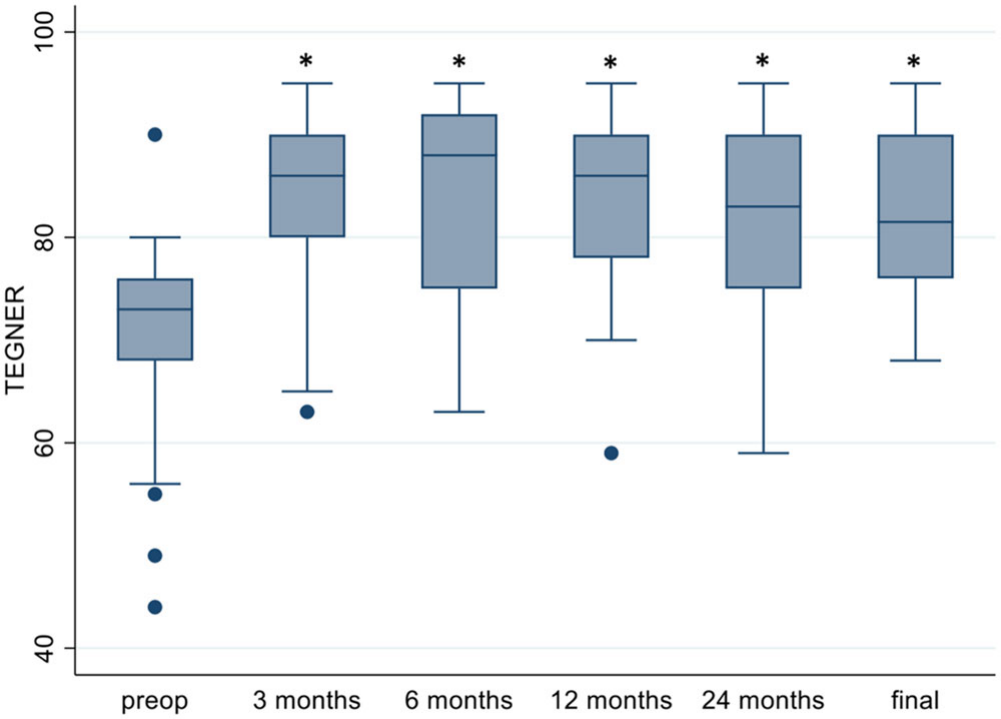
Evaluation of Tegner score 3, 6, 12 and 48 months of follow‐up. On the plot, values from top to bottom are maximum, 75th percentile, median, 25th percentile and minimum. *Statistically significant (*p* < 0.05) compared to baseline.

The average TEGNER score pre‐operatively was 70.9 ± 8.9 and appeared to be marginally increased by a few points to a maximum average of 85.0 ± 8.6 at 3 months, followed by a faint decrease to 82.3 ± 8.0 at the end of the study (Figure 2).

**Figure 3:**
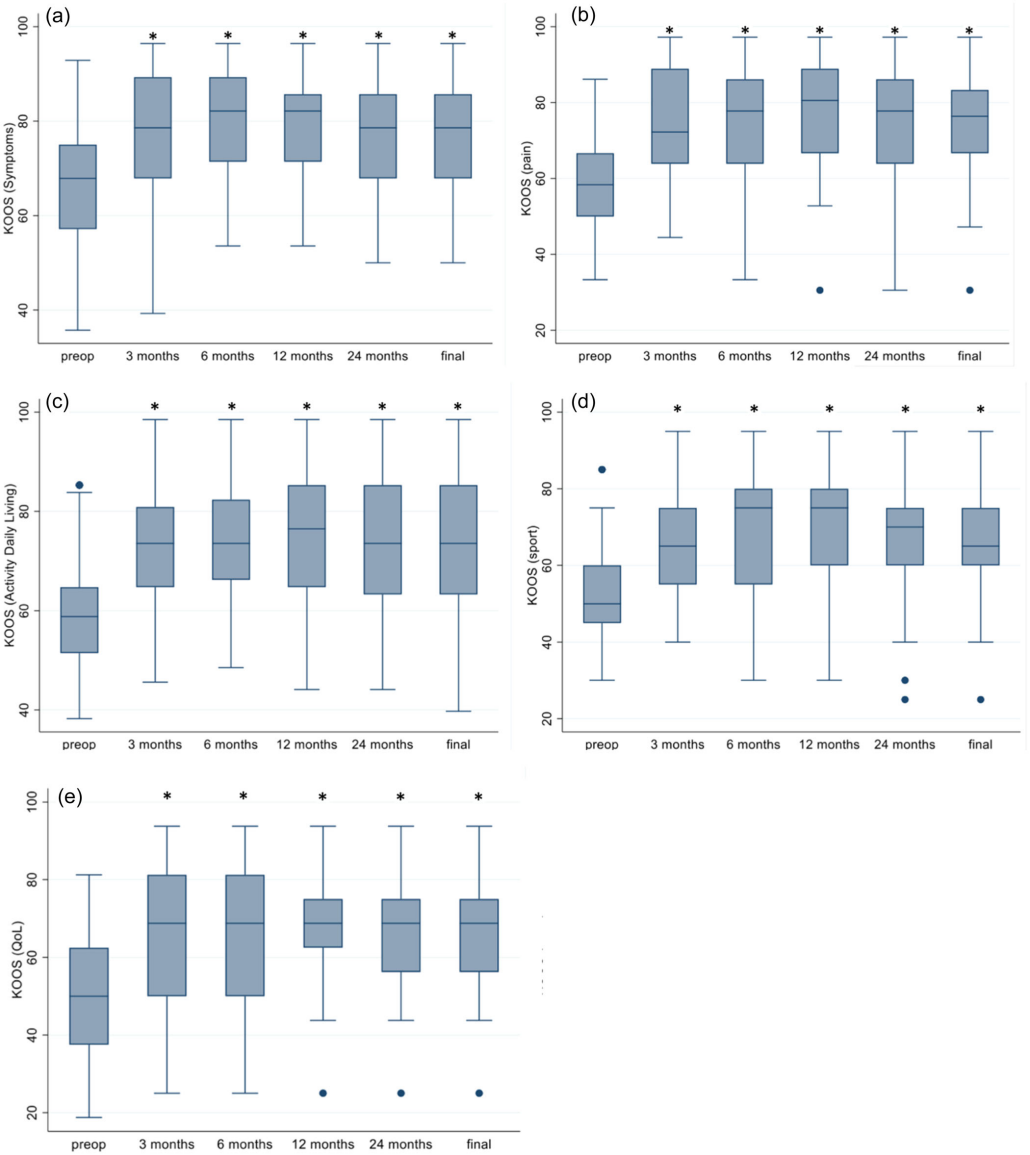
Evaluation of KOOS subscales at 3, 6, 12 and 48 months of follow‐up. On the plot, values from top to bottom are maximum, 75th percentile, median, 25th percentile and minimum. *Statistically significant (*p* < 0.05) compared to baseline. KOOS, Knee Injury and Osteoarthritis Outcome.

All the variations in KOOS subscales (symptoms, pain, activity of daily living, sports, quality of life [QoL]) reached the statistically significant (*p* < 0.05) at 3 months of follow‐up compared to baseline and maintained the improvements up to the last follow‐up, with no further statistically significant variations (Figure 3).

When stratified for age groups, only patients in the group >60 years achieved statistically significant results in all clinical scales, except for KOOS symptoms (Table 2).

**Table 2:** The multilevel mixed‐effects regression analysis represents the change in clinical scores during the follow‐up period. Results were stratified for age groups (<50, between 50 and 60 and >60 years).

	Preop	3 months	6 months	12 months	24 months	48 months	Coeff (95% CI)	*p*
Age ≤ 50								
*N*	16	16	16	15	15	15		
VAS	5.44 ± 1.67	1.75 ± 1.53	1.88 ± 1.75	1.87 ± 1.60	2.40 ± 1.68	2.67 ± 1.63	−0.01 (−0.02 to 0.01)	0.348
TEGNER	73.8 ± 9.6	88.4 ± 8.5	87.5 ± 8.4	86.8 ± 6.9	84.5 ± 6.7	83.9 ± 6.7	0.00 (−0.06; 0.07)	0.919
KOOS symptoms	71.7 ± 15.5	81.7 ± 14.0	81.9 ± 14.7	82.6 ± 14.4	80.2 ± 13.9	80.7 ± 14.7	0.04 (−0.03; 0.10)	0.252
KOOS pain	62.3 ± 12.5	80.6 ± 12.7	80.0 ± 13.6	79.6 ± 14.0	76.7 ± 13.5	76.5 ± 14.3	0.03 (−0.05; 0.11)	0.436
KOOS ADL	64.0 ± 12.0	78.0 ± 11.8	78.1 ± 13.2	78.8 ± 13.1	76.1 ± 13.6	76.6 ± 14.3	0.05 (−0.02; 0.12)	0.152
KOOS sport	57.5 ± 16.5	73.8 ± 15.5	73.1 ± 16.4	73.7 ± 13.7	71.7 ± 14.8	71.7 ± 15.8	0.05 (−0.03; 0.13)	0.218
KOOS QoL	59.0 ± 15.5	73.0 ± 16.1	74.2 ± 14.6	73.3 ± 13.0	70.8 ± 13.7	71.7 ± 15.4	0.04 (−0.04; 0.12)	0.313
50 < Age ≤ 60								
*N*	15	15	15	14	14	13		
VAS	5.60 ± 1.55	2.00 ± 1.20	2.00 ± 2.04	2.07 ± 1.94	2.79 ± 1.97	2.85 ± 1.72	−0.00 (−0.02 to 0.01)	0.699
TEGNER	69.9 ± 8.7	85.3 ± 8.0	86.0 ± 10.3	84.6 ± 11.5	82.0 ± 11.1	83.4 ± 9.3	0.02 (−0.04 to 0.09)	0.502
KOOS symptoms	64.0 ± 10.2	76.2 ± 10.7	77.9 ± 14.2	77.8 ± 11.9	75.0 ± 12.4	75.5 ± 11.2	0.02 (−0.05 to 0.09)	0.600
KOOS pain	60.0 ± 13.1	75.0 ± 12.0	75.6 ± 16.9	78.2 ± 17.3	75.8 ± 17.3	75.4 ± 16.5	0.05 (−0.04 to 0.12)	0.186
KOOS ADL	59.7 ± 14.8	75.2 ± 12.6	76.1 ± 14.2	78.2 ± 14.9	75.2 ± 15.5	74.7 ± 16.5	0.04 (−0.04 to 0.12)	0.309
KOOS sport	50.3 ± 14.2	66.0 ± 13.9	68.7 ± 20.0	68.9 ± 19.6	64.6 ± 19.5	65.4 ± 17.8	0.02 (−0.07 to 0.11)	0.697
KOOS QoL	52.1 ± 12.4	66.7 ± 15.8	67.9 ± 20.7	69.2 ± 18.1	65.6 ± 16.0	65.4 ± 15.4	0.02 (−0.08 to 0.11)	0.704
Age > 60								
*N*	10	10	10	10	10	10		
VAS	6.70 ± 1.49	3.30 ± 1.12	3.10 ± 1.37	3.10 ± 1.45	3.30 ± 1.64	3.30 ± 1.64	−0.02 (−0.04 to −0.00)	0.016
TEGNER	67.8 ± 7.1	79.2 ± 7.1	78.4 ± 6.8	79.1 ± 7.1	78.4 ± 7.4	78.4 ± 7.4	0.07 (0.01–0.13)	0.025
KOOS symptoms	60.0 ± 10.9	72.2 ± 13.9	75.3 ± 11.6	75.7 ± 8.4	70.5 ± 9.4	72.9 ± 7.7	0.09 (−0.02 to 0.20)	0.106
KOOS pain	51.1 ± 11.0	65.0 ± 12.4	69.7 ± 8.0	71.7 ± 10.9	70.0 ± 13.6	69.4 ± 14.0	0.17 (0.05–0.29)	0.004
KOOS ADL	53.8 ± 7.0	64.6 ± 9.5	65.2 ± 7.8	67.4 ± 9.2	67.6 ± 9.4	67.6 ± 9.4	0.13 (0.05–0.21)	0.002
KOOS sport	43.0 ± 11.1	57.5 ± 12.3	60.0 ± 13.3	61.0 ± 11.5	60.5 ± 10.7	60.510.7	0.14 (0.05–0.24)	0.003
KOOS QoL	40.0 ± 12.6	53.1 ± 15.1	56.9 ± 11.2	61.3 ± 10.5	60.6 ± 11.8	60.6 ± 11.8	0.22 (0.09–0.34)	0.001

Abbreviations: ADL, activity of daily living; CI, confidence interval; KOOS, Knee Injury and Osteoarthritis Outcome; QoL, quality of life; VAS, visual analogue scale.

When stratified for KL grade, two groups were formed: mild OA, hence KL I–II (21 patients, of which 2 underwent TKA before 12 months and before the latest follow‐up, respectively) and moderate/severe OA; hence, KL III–IV (20 patients, of which 1 underwent TKA before 12 months). Those with lower KL grades showed statistically significant changes for all categories apart from the Tegner score (Table 3).

**Table 3:** The multilevel mixed‐effects regression analysis represents the change in clinical scores during the follow‐up period. Results were stratified according to Kallgren–Lawrence (KL) in up to mild (1–2) and moderate‐severe (3–4) knee OA classification.

	Preoperative	3 months	6 months	12 months	24 months	48 months	Coeff (95% CI)	*p*
KL 1–2								
*N*	21	21	21	20	20	19		
VAS	5.29 ± 1.55	1.86 ± 1.59	1.86 ± 1.68	1.90 ± 1.62	2.25 ± 1.68	2.26 ± 1.41	−0.01 (−0.02 to −0.00)	0.048
TEGNER	71.5 ± 8.7	86.3 ± 9.4	85.7 ± 9.6	85.2 ± 9.0	83.6 ± 9.2	84.4 ± 7.3	0.04 (−0.01 to 0.10)	0.146
KOOS symptoms	69.6 ± 15.6	80.6 ± 14.8	81.0 ± 15.0	82.1 ± 12.7	80.7 ± 12.1	82.0 ± 11.5	0.07 (0.01–0.12)	0.024
KOOS pain	62.6 ± 12.6	78.8 ± 14.4	79.8 ± 12.9	80.8 ± 11.9	78.9 ± 12.4	80.1 ± 11.7	0.09 (0.02–0.16)	0.011
KOOS ADL	62.2 ± 13.6	75.6 ± 13.5	76.4 ± 13.8	78.2 ± 12.9	76.1 ± 14.0	77.9 ± 13.8	0.09 (0.03–0.15)	0.003
KOOS sport	53.3 ± 16.5	68.8 ± 14.7	70.7 ± 16.1	70.8 ± 15.8	68.8 ± 15.6	70.5 ± 13.9	0.08 (0.01–0.15)	0.028
KOOS QoL	55.1 ± 16.4	69.0 ± 17.7	72.0 ± 17.1	73.1 ± 14.1	70.0 ± 12.9	72.0 ± 12.9	0.09 (0.01–0.17)	0.028
KL 3–4								
*N*	20	20	20	19	19	19		
VAS	6.35 ± 1.57	2.60 ± 1.31	2.60 ± 1.90	2.63 ± 1.80	3.32 ± 1.73	3.53 ± 1.65	−0.01 (−0.02 to 0.01)	0.360
TEGNER	70.3 ± 9.3	83.8 ± 7.7	83.7 ± 9.2	82.8 ± 9.5	80.4 ± 8.3	80.1 ± 8.2	0.01 (−0.04 to 0.06)	0.602
KOOS symptoms	62.3 ± 9.2	74.0 ± 10.3	76.6 ± 12.3	75.9 ± 11.2	70.8 ± 11.3	71.8 ± 10.8	0.02 (−0.05 to 0.08)	0.623
KOOS pain	54.7 ± 12.2	70.4 ± 11.3	71.8 ± 14.5	73.1 ± 16.4	70.2 ± 16.2	68.4 ± 15.7	0.06 (−0.02 to 0.13)	0.148
KOOS ADL	57.6 ± 11.1	71.8 ± 11.5	71.9 ± 12.7	72.9 ± 14.0	71.0 ± 12.9	69.3 ± 13.5	0.04 (−0.03 to 0.10)	0.266
KOOS sport	49.3 ± 14.1	65.0 ± 16.0	65.8 ± 18.9	66.6 ± 16.4	63.7 ± 16.6	62.6 ± 16.7	0.04 (−0.03 to 0.12)	0.289
KOOS QoL	48.4 ± 13.7	62.5 ± 16.5	63.1 ± 17.1	64.1 ± 14.9	62.5 ± 15.2	61.2 ± 15.1	0.06 (−0.02 to 0.14)	0.160

Abbreviations: ADL, activity of daily living; CI, confidence interval; KOOS, Knee Injury and Osteoarthritis Outcome; OA, osteoarthritis; QoL, quality of life; VAS, visual analogue scale.

The only post‐operative complication reported was the abdominal haematoma observed in 11 patients which self‐resolved within the first month.

The failure rate was assessed as well. Three patients underwent knee replacement surgery during the time of the study, specifically two patients before 12 months and one patient after 24 months of follow‐up time point.

## DISCUSSION

The main finding of the present prospective clinical trial is that a single intra‐articular injection of micro‐fragmented adipose tissue following knee arthroscopic debridement offers significant amelioration in clinical scores and a low failure rate up to 4 years of follow‐up.

The rationale behind the present study lies in the potential of MSCs in restoring and regenerate damaged tissues [29]. MSCs are naturally found in most vascularized tissue, although the most common harvesting sites in clinical practice are adipose tissue, bone marrow or foetal annexes [16, 28]. Despite some attempts to establish the optimum in terms of the quality of the tissue; nowadays, the available literature is not unanimous on the best harvesting site [19, 44]. However, in vitro studies demonstrated that adipose tissue can contain up to 300‐fold the number of MSCs when compared to the same volume of bone marrow aspirate [4, 31]. Moreover, adipose tissue harvesting is considered an easy and reproducible procedure by most surgeons, with a low intraoperative complication rate and an even lower rate of post‐operative adverse events [25, 40, 41]. In our study, we reported no complications related to the procedure except for a minimal abdominal haematoma which self‐resolved in few days; furthermore, the minimal invasiveness of the procedure allowed the same‐day discharge with no post‐operative adverse events reported. This can be considered the direct consequence of the advent of one‐step procedures in the field of minimally manipulated products [15]. In fact, given the restrictions currently applied in the field of manipulation of cells outside the operating room, the most common use of these products is possible thanks to regulatory shortcuts such as ‘501k exemption’, which allows to introduce in the market of new commercial kits if equivalent to those already in use, hence waiving the FDA approval [15, 22]. The clear advantage of a one‐step procedure is however counterbalanced by the lack of standardization and the impossibility of a clear quality assessment of the tissue harvested. Nonetheless, beyond those limits, the role of MSCs for OA has been extensively proven to counteract inflammatory status and eventually promote regeneration of the tissues [10] and data regarding both preclinical and clinical effects are starting to build strong scientific evidence. In vitro studies demonstrated how MSCs co‐cultured with human chondrocytes were able to stimulate proliferation and collagen formation [35]. Furthermore, clinical outcomes collected by a recent meta‐analysis testified to the safety and good clinical outcomes of adipose‐derived mesenchymal cells for the treatment of knee OA [1]. When addressing specific preparation kits, such as the Lipogems® used in the present study, clinical results found in the literature are comparable to the results of our trial. In particular, Bistolfi et al. reported that intraarticular autologous adipose tissue injection in patients with knee OA, KL Grades I–III, reduced knee pain and stiffness. Patients gained improved knee function and experienced a better QoL in daily and sports activities with the absence of severe complications [8]. The average follow‐up was 23.5 months, shorter than ours, although they presented a pool of 87 patients versus ours consisting of only 41 patients. Moreover, Schiavone Panni et al. also demonstrated an improvement in knee function scores and VAS pain scale on 52 patients with a mean follow‐up length of 15.3 months [39]. The mean International Knee Society scoring system function improved from 37.4 pre‐operatively to 62.6 and the mean VAS decreased from 8.5 to 5.1 (134). It has also been observed that patients with VAS scores greater than 8 showed greater clinical and functional benefits as compared to those with VAS lower than 8. Barfod et al. also reached similar results as those obtained by our study. They followed up with 20 patients for up to 12 months on whom the Lipogems® system was used. The authors determined a statistically significant improvement in the KOOS for all subscales [6]. Comparative studies of the micro‐fragmented adipose tissue injections with other injective options were also carried out, showing the potential of this treatment alternative. Richter et al. randomized 75 knee OA patients into three groups, consisting of corticosteroid injection, micro‐fragmented adipose tissue injection and saline injection. Clinical evaluation through Western Ontario and McMaster University Osteoarthritis Index and KOOS scores were conducted up to 1 year of follow‐up. The micro‐fragmented adipose tissue group demonstrated superiority compared to placebo up to 1 year, whereas the corticosteroids group was statistically significantly superior to placebo only up to 6 weeks of follow‐up [38]. Zaffagnini et al. conducted a randomized controlled trial treating with micro‐fragmented adipose tissue injection or platelet‐rich plasma injection (PRP) with a total of 118 patients with symptomatic knee OA. They found statistically significant improvements in all clinical scores assessed up to 2 years of follow‐up, although no significant difference was found in terms of clinical or radiological response between the two treatment options. Interestingly, statistically significantly more patients in the micro‐fragmented adipose tissue group with moderate/severe OA reached the minimal clinically important difference for the International Knee Documentation Committee score at 6 months compared with the PRP group [43]. Given the above‐mentioned scattered literature on the topic, we believe that the major strength of our study is the longer follow‐up period, being to our knowledge the first study in this setting to have assessed patients up to 48 months. Moreover, conducting a concomitant arthroscopy enabled us to evaluate the presence of disease different from OA which may have caused the symptoms. We are well aware of how a concomitant procedure apart from the intra‐articular injection could potentially offer a source of bias; however, this might be of minimal relevance considering how recent evidence is proving how the arthroscopy alone is not sufficient to provide symptom relief in knee OA [30].

Furthermore, we believe that a further strength of this study is the stratification of the patients. When stratifying by age, patients older than 60 years achieved better clinical results compared to younger patients and this could be a game‐changer finding considering that this is usually the cluster of patients to whom a knee replacement is more often proposed. Conversely, statistical significance was obtained for patients with less severe radiological OA, suggesting that MSCs could better exert their role on functional outcomes when symptoms are caused by inflammation rather than bone‐to‐bone contact, confirming their significant anti‐inflammatory effect.

Nonetheless, this study suffers from several limitations. First, the limited sample size and the absence of a preliminary power calculation severely limit the impact of our findings. Accordingly, the lack of a control group is responsible for a confounding bias, since it is not possible to assess if the retrieved results are the consequence of our intervention or external factors. Furthermore, the absence of post‐operative imaging of any type can circumscribe our finding to a mere symptomatic relief and we were not able to assess any potential disease‐modifying effects on the disease progression.

## CONCLUSIONS

A single intra‐articular knee injection of micro‐fragmented adipose tissue following arthroscopic debridement was able to provide significant clinical benefits in patients affected by knee OA for up to 4 years of follow‐up.

## AUTHOR CONTRIBUTIONS

Andrea Antonio Maria Bruno, Enrico Arnaldi and Elizaveta Kon contributed to the study conception and design. Material preparation, data collection and analysis were performed by Massimo De Donato, Marco Basso, Jacopo Tamini and Paolo Dupplicato. The first draft of the manuscript was written by Andrea Antonio Maria Bruno. Giuseppe Anzillotti and Berardo Di Matteo contributed to the writing of the first draft. All the other authors conducted a critical review and editing of the final draft. All authors read and approved the final manuscript.

## CONFLICT OF INTEREST STATEMENT

Elizaveta Kon is a consultant for Zimmer Biomet, Smith & Nephew, GreenBone, Geistlich Pharma, Fidia Farmaceutici, Mastelli and TerraQuantum, all not related to the present article. The remaining authors declare no conflicts of interest.

## ETHICS STATEMENT

The internal Humanitas Research Hospital ethical committee approved the present study (study no. 2219). Written informed consent was obtained for all the patients included in the present study.

## Data Availability

All the retrieved results are presented in the published paper. Raw data can be asked to the corresponding author, upon reasonable request.
